# Functionalized AIE nanoparticles with efficient deep-red emission, mitochondrial specificity, cancer cell selectivity and multiphoton susceptibility†
†Electronic supplementary information (ESI) available: Further details of TPE-TETRAD synthesis and characterization. See DOI: 10.1039/c7sc00908a



**DOI:** 10.1039/c7sc00908a

**Published:** 2017-05-09

**Authors:** Alexander Nicol, Wei Qin, Ryan T. K. Kwok, Jeffrey Mark Burkhartsmeyer, Zhenfeng Zhu, Huifang Su, Wenwen Luo, Jacky W. Y. Lam, Jun Qian, Kam Sing Wong, Ben Zhong Tang

**Affiliations:** a Division of Biomedical Engineering , Department of Chemistry , Hong Kong Branch of Chinese National Engineering Research Center for Tissue Restoration and Reconstruction , Institute for Advanced Study , Institute of Molecular Functional Materials , State Key Laboratory of Molecular Neuroscience , The Hong Kong University of Science and Technology (HKUST) , Clear Water Bay , Kowloon , Hong Kong , China . Email: tangbenz@ust.hk; b HKUST-Shenzhen Research Institute , No. 9 Yuexing 1st RD, South Area, Hi-tech Park Nanshan , Shenzhen 518057 , China; c Department of Physics , HKUST , Clear Water Bay , Kowloon , Hong Kong , China; d State Key Laboratory of Modern Optical Instrumentation , Centre for Optical and Electromagnetic Research , Zhejiang Provincial Key Laboratory for Sensing Technologies , Zhejiang University , 310058 Hangzhou , China; e Guangdong Innovative Research Team , SCUT-HKUST Joint Research Laboratory , State Key Laboratory of Luminescent Materials and Devices , South China University of Technology , Guangzhou 510640 , China

## Abstract

Multiphoton microscopy is an exciting tool for biomedical research because it can be used to image single cells *in vivo* due to its greater penetration depth, lower phototoxicity and higher resolution when compared to confocal laser scanning microscopy. This helps researchers understand how certain cells change over time and evaluate the efficacy of different therapies. Herein, we report a new AIE luminogen (AIEgen), abbreviated as TPE-TETRAD, with a favorable absorption and efficient deep-red emission in the solid state. TPE-TETRAD possesses a high two-photon absorption cross-section (313 MG at 830 nm) and a rich array of non-linear optical properties including aggregation-induced three-photon luminescence. Biotinylated TPE-TETRAD nanoparticles are also fabricated and applied to stain mitochondria in live cancer cells with high specificity. The purpose of this study is to characterize a novel deep-red AIEgen and fabricate biotinylated nanoparticles for applications as (1) biocompatible and photostable AIE probes for specific mitochondria imaging and (2) multiphoton imaging probes suitable for two/three-photon fluorescence microscopy.

## Abstract 2

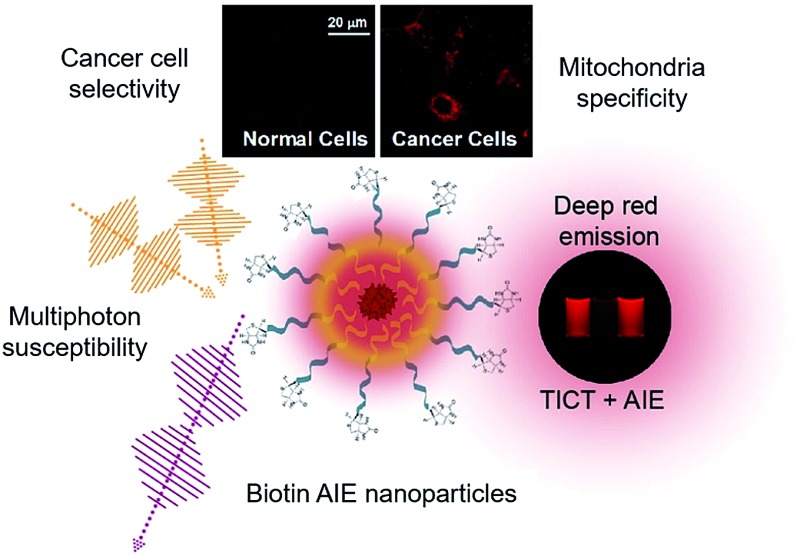
Mitochondria targeting biotinylated AIE nanoparticles are used as multiphoton imaging probes to identify cancer cells.

## Introduction

Fluorescent imaging agents have proven themselves to be an important enabling technology for modern biology, much like how the advent of the optical microscope enabled early microbiologists like Anton van Leeuwenhoek to look beyond tissue and observe single cells. Fluorescent proteins,^1–3^ organic dyes^4,5^ and nanoparticles (NPs)^6–8^ have all played a pivotal role in the non-invasive study of gene expression,^9^ protein function,^10^ protein–substrate interactions^11,12^ and many other cellular processes.^13–15^ In particular, fluorescent dyes with emission wavelengths in the deep red to near-infrared region are highly desirable for *in vivo* bioimaging as the surrounding tissue will exhibit lower optical absorption and weaker interference from autofluorescence.^16–18^ By using red fluorophores, it is possible to obtain a higher degree of tissue penetration and resolution than by using imaging agents with bluer emission.^19,20^ To date, the most widely used commercial red fluorescent probes are organic fluorophores.^21^ One such prototypical example is dicyanomethylene-4*H*-chromene (DCM) and its many reported derivatives.^22^ DCM intrinsically has an emission maximum above 600 nm but this can be further red-shifted through the addition of electron-donating groups, creating a favorable donor–acceptor (D–A) system.^23,24^ However, this increase in synthetic complexity has also been shown to negatively affect the processability, water solubility and cell membrane permeability.^25^ Furthermore, the highly planar structure and poor water solubility of DCM derivatives lead to aggregate formation in biological media due to strong π–π interactions. In the aggregated state, decreased emission intensity is often observed in DCM derivatives due to the aggregation-caused quenching (ACQ) and twisted intramolecular charge transfer (TICT) properties of the D–A system.

**Scheme 1:**
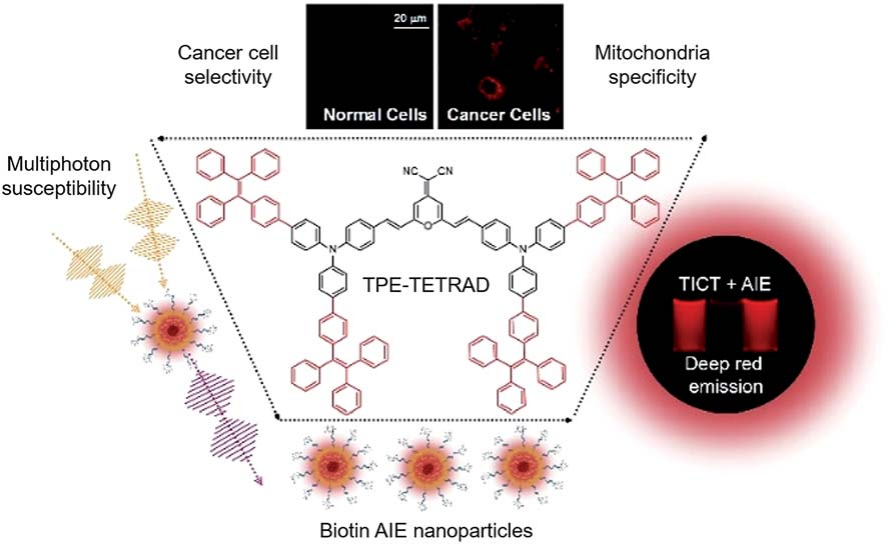
Highlighted features and applications of TPE-TETRAD.

A seminal work by our research group found that engineering new molecules with propeller-shaped and rotatable conjugated rings led to novel photophysical properties which contradicted those of the ACQ effect.^26^ Unlike conventional systems, these fluorophores are highly emissive when aggregated due to a process now known as aggregation-induced emission (AIE).^27,28^ Tetraphenylethene (TPE) is one of the most iconic and widely used AIE building blocks. However, the majority of AIE fluorophores incorporating TPE are blue and green emitters because they lack electron transfer properties.^29,30^ Despite the consistently high fluorescence quantum yield of TPE-based AIE systems, *in vivo* biological applications cannot be realized due to their short emission wavelength, which overlaps with cell autofluorescence.^31^ A recent molecular engineering principle formulated in our group has shown that modifying existing ACQ fluorophores like DCM with AIE building blocks like TPE will lead to efficient solid state emission.^32,33^ Combining DCM derivatives with TPE moieties serves the dual roles of extending the π-conjugation of the molecule to further red-shift its emission and changing it into a non-coplanar structure with AIE properties. Following this molecular design principle, we plan to synthetically modify a DCM derivative 2-(2,6-bis((*E*)-4-(diphenylamino)styryl)-4*H*-pyran-4-ylidene)malononitrile (TPA-DCM) with four TPE AIE units to yield 2-(2,6-bis((*E*)-4-(bis(4′-(1,2,2-triphenylvinyl)-[1,1′-biphenyl]-4-yl)amino)styryl)-4*H*-pyran-4-ylidene)malononitrile (TPE-TETRAD), as emphasized in Scheme 1. The term “TETRAD” highlights the fact that there are four TPE groups arranged as a tetrad pair on the TPA-DCM core.

Based on our previous experience, we expect that this large and hydrophobic AIE molecule would have very low cell-permeability *via* passive diffusion or endocytosis.^34,35^ Previous reports have enhanced the cellular uptake of AIEgens using nanoparticles (NPs) coated with peptides,^36^ antibodies^37^ and biomolecules.^32,38–41^ Each of these techniques varies significantly in its target selectivity, fabrication complexity and cost. In order to enhance the cellular uptake of TPE-TETRAD to realize bioimaging applications, DSPE–PEG_2000_–biotin was selected to encapsulate TPE-TETRAD to make uniform spherical AIE NPs (TPE-TETRAD@biotin). Biotin PEG was selected because it had balanced advantages in selectivity, complexity and cost. Moreover, our literature study suggested that biotin could be used to specifically stain the mitochondria of cancer cells due to the unique receptor-mediated endocytosis pathway of the avidin–biotin interaction.^42^ Previous studies have shown that biotin (vitamin B7) is a growth promoter important for cancer cell proliferation.^43^ Accordingly, cancer cells have more avidin–biotin binding sites on their cell surface when compared to normal cells.

In theory, this should translate into greater uptake of the TPE-TETRAD@biotin NPs by cancer cells and consequently allow for their identification.^44^ Furthermore, the mitochondria contain many endogenous biotinylated proteins which are important regulators for amino acid catabolism.^45^ Consequently, there are known receptor-mediated endocytosis pathways linking biotin trafficking to the mitochondria which may allow for the targeted delivery of TPE-TETRAD@biotin NPs into the mitochondria.^46^ Herein, we report TPE-TETRAD@biotin NPs that were able to preferentially stain the tubular mitochondrial structures of HeLa cervical cancer cells. The specificity of these AIE NPs to cancer cells makes them powerful diagnostic tools that may aid in advancing medical therapies such as fluorescence image-guided surgery.^47^ To the best of our knowledge, this is the first demonstration of deep-red AIE NPs for mitochondria specific imaging in live cancer cells.^48^

Deep-red or near-infrared emission has been shown to be useful for increasing the penetration depth for optical imaging techniques *in vivo*. However, there is a limit to this approach and reports have rarely exceeded more than several micrometers *in vivo*.^49^ To achieve optical *in vivo* penetration depths up to 1000 μm, two-photon or multiphoton microscopy must be used.^50^ In the past decade, advances in optical techniques have shown that the excitation of some organic materials with femtosecond laser pulses can yield three-photon absorption (3PA) and four-photon absorption (4PA).^51^ These discoveries have pushed the frontiers of bioimaging because with multiphoton imaging, a longer wavelength of coherent light could be used. This is in addition to the fact that there is a non-linear dependence of multiphoton excitation probability on the local intensity of the applied light field.^52^ Previous reports have shown that several DCM derivatives with D–A structures possess favorable two-photon absorption cross-sections (2PA).^53^ A recent study reported AIE molecules which exhibited two-, three- and even four-photon excited fluorescence (2PL/3PL/4PL) and other unique non-linear optical properties.^54,55^ TPE-TETRAD is also expected to show similar 2PL/3PL properties because of its large π-electron delocalization and D–A structure. As there are relatively few reports of AIE NPs that combine the unique advantages of deep-red emission, mitochondrial specificity, high photostability, cancer cell identification and multiphoton imaging, we hope that our attempt to successfully demonstrate these will inspire more exciting research into this emerging interdisciplinary field of AIE-based bioimaging and diagnostics.

## Results and discussion

### Characterization of TPE-TETRAD

**Scheme 2:**
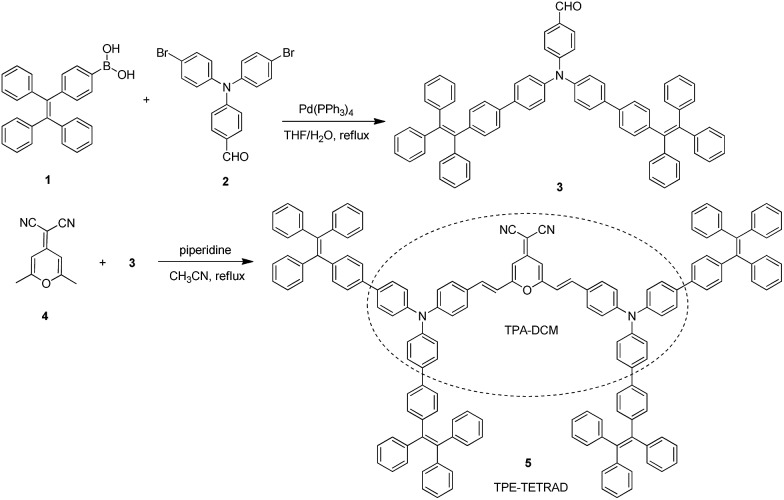
Synthetic route to TPE-TETRAD.

TPA-DCM was prepared according to our previous report^32^ and TPE-TETRAD was prepared according to the synthetic route shown in Scheme 2. The purified products were characterized using standard spectroscopic methods including ^1^H NMR, ^13^C NMR and high resolution mass spectrometry, which confirmed their correct structures (see ESI, Fig. S1–S3†).

TPE-TETRAD consists of a TPA-DCM core (black dashed circle) and four TPE pendent groups connected through single bonds as shown in Scheme 2. TPA-DCM consists of two TPA donor (D) units and one DCM acceptor (A) unit forming a typical donor–π–acceptor–π–donor (D–π–A–π–D) structure. The addition of four TPE units serves to strengthen the donor groups, extend the π-conjugation and endow the molecule with AIE properties.

**Fig. 1:**
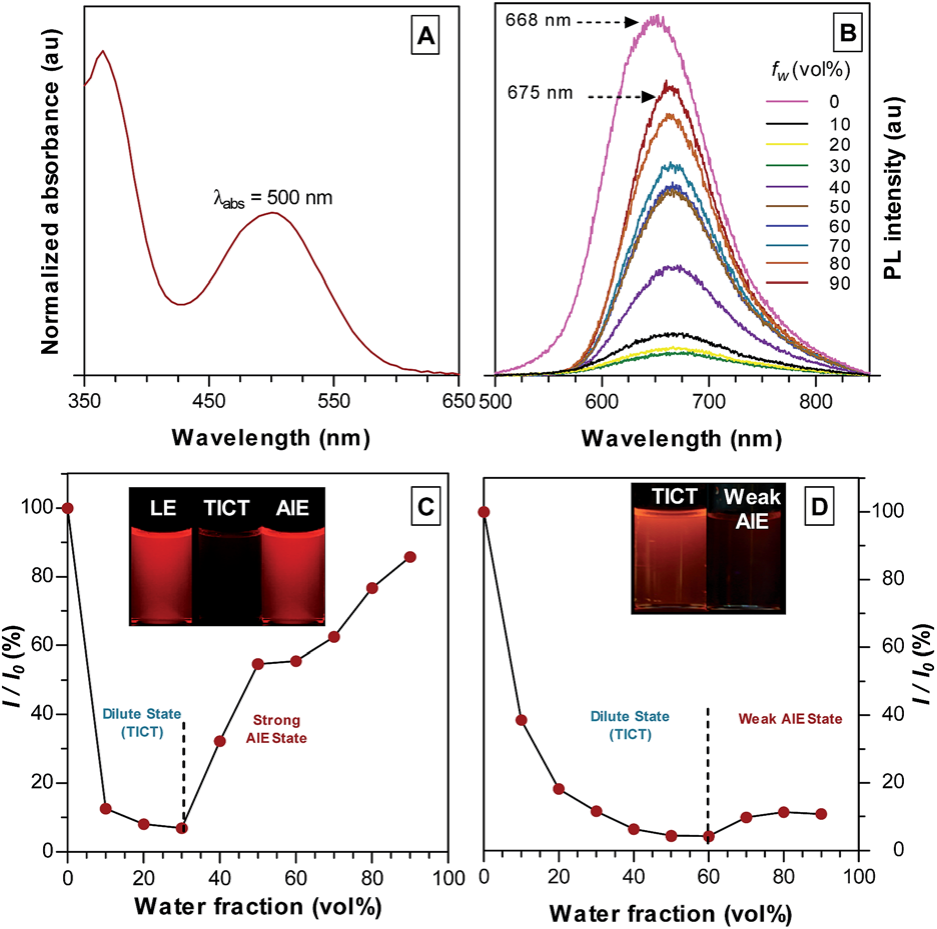
(A) Normalized UV-Vis absorption spectrum of TPE-TETRAD in THF. (B) PL spectra of TPE-TETRAD in THF/water mixture with different water fractions (*f*_w_). (C) Plot of relative PL intensities (*I*/*I*_0_) *versus f*_w_, where *I*_0_ is the maximum PL intensity of TPE-TETRAD in THF at 668 nm and *I* is the fluorescence intensity after each subsequent scan. Inset: images of LE state (100%), dilute TICT state (10%) and AIE state (90%) emission taken under a UV lamp with 355 nm excitation. Conditions: [TPE-TETRAD] = 10 μM; *λ*_ex_ = 500 nm. (D) Plot of PL intensities (*I*/*I*_0_) *versus f*_w_, where *I*_0_ is the maximum PL intensity of TPA-DCM in THF at 620 nm and *I* is the fluorescence intensity after each subsequent scan. Inset: images of dilute state (0%) and aggregate state (90%) emission taken under a UV lamp with 355 nm excitation. Conditions: [TPA-DCM] = 10 μM; *λ*_ex_ = 465 nm.

The optical and structural properties of TPA-DCM and TPE-TETRAD are summarized in Table S1 and Fig. S4–S6 in the ESI.† TPA-DCM in THF has an absorption maximum at 465 nm with a molar absorptivity of 7.1 × 10^4^ L mol^–1^ cm^–1^. The photoluminescence (PL) of TPA-DCM was measured in a THF/water mixture with different water fractions (*f*_w_). This experiment allowed for a controlled analysis of solute induced aggregation, which directly allowed us to determine the optical properties of TPA-DCM. In pure THF solution, TPA-DCM showed an intense red fluorescence and an emission maximum at 620 nm. When the water fraction was increased, the emission dropped dramatically and bathochromically shifted to 670 nm (Fig. 1D). This phenomenon is typical of many fluorophores with TICT properties, where the emission is red-shifted and weakened in high polar solvents. A slight increase in emission was observed when the water fraction was over 60%, indicating that TPA-DCM is a weakly AIE but predominantly TICT system. Although TPA-DCM is a TICT fluorophore, it possesses many favorable optical properties for bioimaging, including its deep-red emission and high photostability. In an effort to gain strong aggregate emission, we followed a structural design principle^56^ which allowed us to modify the existing structure of TPA-DCM with AIE building blocks like TPE to yield efficient solid state emission. The resulting molecule TPE-TETRAD was formed by the synthetic addition of four TPE units to form a D–π–A–π–D structure. The absorption peak of TPE-TETRAD in THF is 500 nm, which is ∼35 nm red-shifted compared to TPA-DCM, as shown in Fig. 1A. The absorption of TPE-TETRAD is well matched to conventional gas laser wavelength sources, which typically range between 458–514 nm, making it ideal for confocal and multiphoton microscopy. TPE-TETRAD exhibits an emission maximum at 668 nm in THF, which is ∼50 nm red-shifted from that of TPA-DCM. TPE-TETRAD has a relatively large Stokes shift of 168 nm.

The photophysical properties of TPE-TETRAD are a combination of both strong AIE and TICT characteristics as shown in Fig. 1C. The locally-excited (LE) state is highly emissive in pure THF but its emission dramatically decreases as the solvent polarity is increased by adding water. At the 30% water fraction, the AIE state starts to dominate and the emission is further intensified. The emission wavelength of TPE-TETRAD is bathochromically shifted from 668 to 675 nm during the solvent-induced aggregation process due to the competing TICT-AIE forces (Fig. 1B).

### Fabrication and characterization of TPE-TETRAD@biotin NPs

**Scheme 3:**
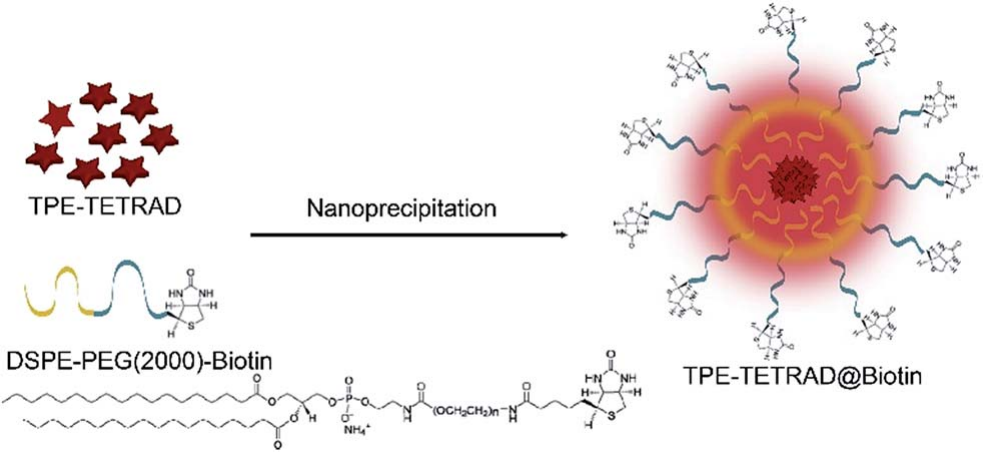
Schematic illustration of TPE-TETRAD@biotin NP fabrication.

The efficient aggregate emission of TPE-TETRAD prompted us to explore its bioimaging applications. TPE-TETRAD aggregates were prepared and incubated with HeLa cells. However, no cellular uptake or emission was found after several attempts with varying incubation period. The lack of TPE-TETRAD aggregate uptake may be due to its hydrophobic surface chemistry. In order to facilitate the cellular uptake of TPE-TETRAD, biotinylated AIE NPs (abbreviated as TPE-TETRAD@biotin) were fabricated using a modified nanoprecipitation method as shown in Scheme 3.^57^ TPE-TETRAD and DSPE–PEG_2000_–biotin were first dissolved in THF and stirred in a scintillation vial to form a homogeneous red mixture. The THF was completely dried and then the solid was redispersed in Milli-Q water *via* sonication.

**Fig. 2:**
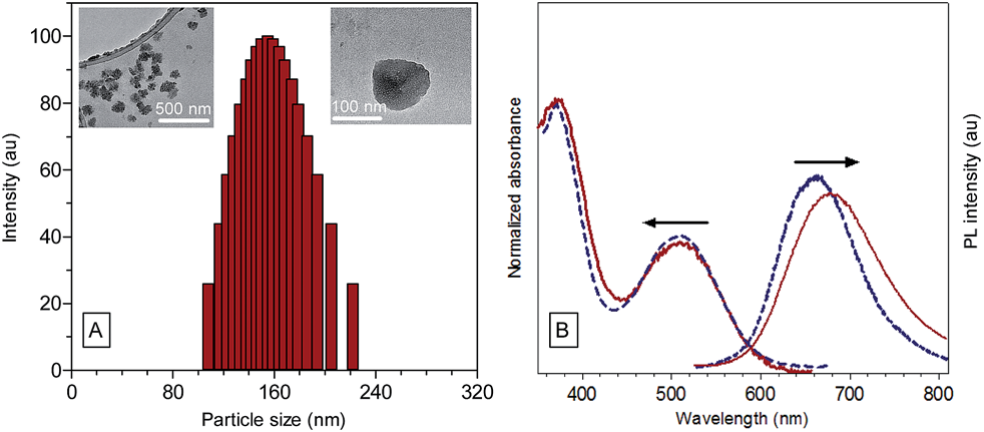
(A) Particle size distribution of TPE-TETRAD@biotin NPs measured by dynamic light scattering, showing a mean hydrodynamic diameter of 155 nm with a narrow polydispersity of 0.05 PDI. Inset: TEM images of the AIE NPs with different scale bars. (B) Normalized UV-Vis absorption and PL emission spectra of the AIE NPs (red; *λ*_abs_ = 510 nm, *λ*_em_ = 680 nm) and TPE-TETRAD nanoaggregates (blue; *λ*_abs_ = 500 nm, *λ*_em_ = 675 nm) in water. [TPE-TETRAD] = 10 μM; [TPE-TETRAD@biotin] = 6 μg mL^–1^; *λ*_ex_ = 488 nm.

The suspension was then filtered using a 0.45 μm filter to afford a stock TPE-TETRAD@biotin NP solution with a dried mass of roughly 0.25 mg and a concentration of 250 μg mL^–1^, or 0.8 nM. A calculation of the TPE-TETRAD@biotin stock and working concentration is provided in the ESI.† The average hydrodynamic diameter for the AIE NPs was measured to be 155 nm by dynamic light scattering (DLS) with a low polydispersity index (PDI) of 0.05, as shown in Fig. 2A. This size is ideal to induce the enhanced permeability and retention (EPR) process, which will lead to greater accumulation in cancer cells and increased bioimaging efficiency. TPE-TETRAD@biotin NPs were chemically and colloidally stable for several weeks when kept in a sealed scintillation vial at room temperature, suggesting that they can stay well dispersed in aqueous solution as single NPs without forming large aggregates. This was further confirmed by the spherical NP shape in the transmission electron microscopy (TEM) images shown in Fig. 2A. Absorption and emission spectra of the TPE-TETRAD@biotin NPs were measured immediately once the NPs were fabricated. As shown in Fig. 2B, the TPE-TETRAD nanoaggregates in aqueous solution showed two absorption maxima at 365 and 500 nm, while the spectra of the AIE NPs showed slightly red-shifted absorption peaks at 375 and 510 nm. The emission maxima wavelengths were 675 and 680 nm for the TPE-TETRAD nanoaggregates and TPE-TETRAD@biotin NPs, respectively. The excitation and emission profiles of TPE-TETRAD@biotin match well with commercial laser sources and common fluorescence-imaging systems, making it promising for bio-imaging applications. The photophysical properties of TPE-TETRAD in different states were investigated and reported in Table 1. The quantum yield (*η*) was the greatest for TPE-TETRAD in the solid-state thin film as compared to the isolated molecules in THF, nanoaggregates in water and TPE-TETRAD@biotin NPs. This can be attributed to the highly favorable solid-state emission features of AIE luminogens like TPE-TETRAD. The encapsulation efficiency, dye loading ratio and quantum yield of the TPE-TETRAD@biotin NPs were optimized based on our previous report.^32^ However, the *η* of the AIE NPs (*η* = 12%) was still much lower than that in the solid state (*η* = 23%). This can be attributed to solvent quenching effects from the surrounding water molecules and possibly different molecular packing arrangements of TPE-TETRAD molecules in the solid state *versus* the NP state.

**Table 1:** Photophysical properties

	Solution^*a*^	Aggregates^*b*^	Solid-state^*c*^	NPs^*d*^
Quantum yield (*Φ*, %)^*e*^	20.94	20.78	23.47	12.52
Lifetime (*τ*, ns)^*f*^	3.97	1.55	3.55	4.12
Emission max (*λ*_em_, nm)^*g*^	668	675	665	680

^*a*^TPE-TETRAD in pure THF solution, 10 μM.

^*b*^Aggregates in THF/water 1 : 9 v/v, 10 μM.

^*c*^Thin film on glass cover slide.

^*d*^TPE-TETRAD@biotin NPs in water, 6 μg mL^–1^.

^*e*^Quantum yield measured using an integrating sphere.

^*f*^Time-domain method.

^*g*^Emission maximum.

The relationship between the fluorescence quantum yield (*η*) and the fluorescence lifetime (*τ*) is given by eqn (1) and (2) below, where *Γ* is the radiative decay term and *k*_nr_ is the non-radiative decay term.
1

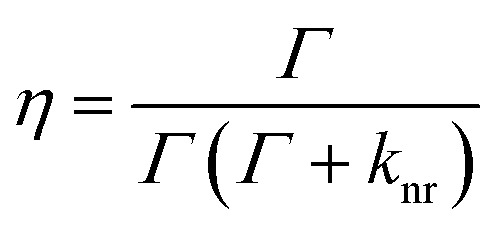



2

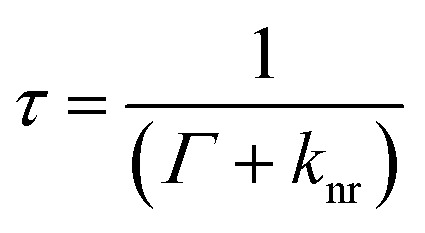




The radiative decay term (*Γ*) is an intrinsic property of the fluorophore which can be treated as a constant. Therefore, when examining eqn (1) and (2), the non-radiative decay term (*k*_nr_) is generally regarded as the variable factor in determining the fluorescence quantum yield (*η*) and the lifetime (*τ*). The fluorescence lifetime values are listed in Table 1 and their corresponding fluorescence lifetime decay curves are shown in Fig. S7, ESI.† The longest lifetime was observed for the TPE-TETRAD@biotin NPs (see ESI, Fig. S7C†) followed by the molecules in THF (see ESI, Fig. S7D†), solid state (see ESI, Fig. S7A†) and aqueous nanoaggregates (see ESI, Fig. S7B†). It should be noted that the average fluorescence lifetime of the TPE-TETRAD aggregates in H_2_O is less than half of the relative lifetimes of the other three samples tested. The shorter lifetime for the TPE-TETRAD nanoaggregates can be explained by considering the large direct contact area between TPE-TETRAD and water/oxygen molecules, which are known fluorescence quenching agents that will decrease *τ*. The PEG polymer surrounding the AIE NPs is believed to prevent direct contact between TPE-TETRAD and water/oxygen molecules, which is the reason why a relatively longer *τ* of 4.12 ns was observed.

### Biological applications of TPE-TETRAD@biotin NPs

A critical consideration for achieving biological applications includes understanding the cytotoxicity of the AIE NPs. This comprises not only the AIE NPs’ intrinsic toxicity but also the added toxicity that can be generated when the AIE NPs are excited by a high powered laser during fluorescence imaging, which has been reported to generate high levels of reactive oxygen species (ROS) in similar sized aromatic photosensitizers.^58^ On one hand, the generation of ROS can be beneficial if one desires the targeted cell to be killed.^59^ However, it is often the case that one desires long-term cell tracking with limited cytotoxicity and high photostability. This is particularly relevant for two-photon excitation fluorescence (TPEF) microscopy, where long laser exposure is required to build up a complete image scan. ROS generation occurs as a result of intersystem crossing from the singlet excited state to the triplet state upon excitation of a photosensitizing molecule.^60^ The triplet state may subsequently react with molecular oxygen to yield ROS including singlet oxygen (^1^O_2_) species. A longer triplet state lifetime has been shown to increase the likelihood of a reaction with molecular oxygen to form ROS. ^1^O_2_ species are extremely toxic to critical cellular functions, leading to DNA damage and irreversible organelle failure.

**Fig. 3:**
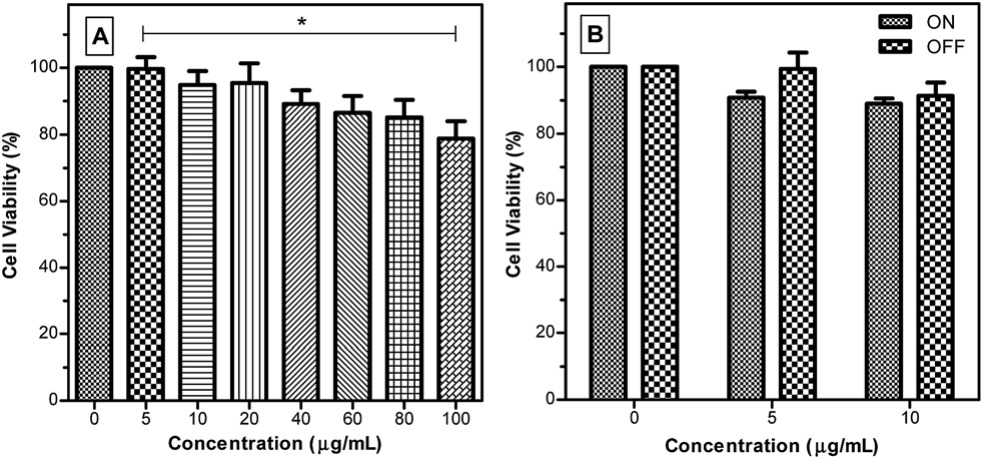
Cell viability assay of HeLa cervical cancer cells after incubation with different TPE-TETRAD@biotin NP concentrations for (A) 24 h and (B) 24 h at 37 °C with variable light exposure (ON/OFF) to quantify ROS cytotoxicity. Asterisk indicates a significant difference between trials.

An MTT assay, a colorimetric assay for assessing cell metabolic activity, was used to evaluate the cytotoxicity of TPE-TETRAD@biotin to HeLa cervical cancer cells in a concentration range of 0–100 μg mL^–1^. Cell viability rates were above 95% at the working concentration of 6 μg mL^–1^ (Fig. 3A). Even at 100 μg mL^–1^ the cell viability was around 80%, indicating that TPE-TETRAD@biotin is biocompatible. To evaluate the possible added cytotoxicity due to ROS generation, a 2′,7′-dichlorofluorescin diacetate (DCFH-DA) cellular ROS detection assay kit was used to characterize the possible ROS generation from TPE-TETRAD@biotin. It was found that the ROS generation of TPE-TETRAD@biotin was not significantly different to that of the control group (see ESI, Fig. S8†). To confirm this result *in vitro*, another MTT assay set was performed. One group was incubated under intense visible light whereas the other group was incubated under normal dark conditions.

These two groups were also exposed to several serial doses of TPE-TETRAD@biotin (0–100 μg mL^–1^), as shown in Fig. 3B. An analysis of variance (ANOVA) test was conducted at a 95% confidence interval (*P* < 0.05) in order to assess whether the light treatment affected cell viability and whether the light treatment had the same effect at all the concentrations. The answer to both questions was no, suggesting that the little (if any) ROS generation from the AIE NPs was not significantly cytotoxic.

**Fig. 4:**
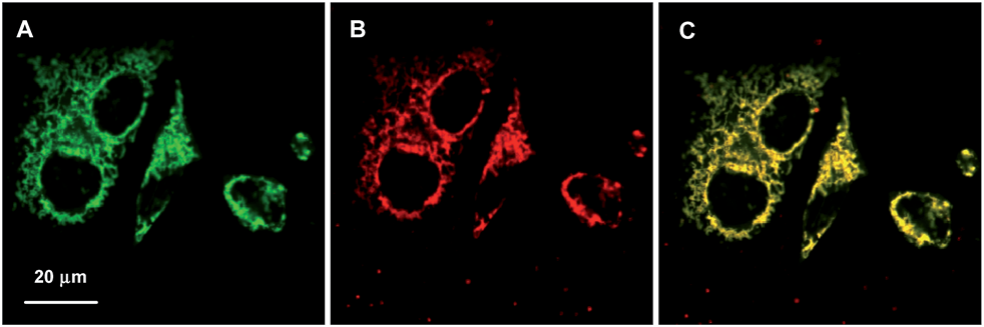
CLSM images of HeLa cervical cancer cells incubated with (A) 25 nM MitoTracker Green (*λ*_ex_ = 488 nm, 500–580 nm emission filter) for 10 min and (B) 6 μg mL^–1^ TPE-TETRAD@biotin NPs (*λ*_ex_ = 488 nm, 600–750 nm emission filter) for 1 h. (C) The merged images of (A) and (B).

The bioimaging applications of the TPE-TETRAD@biotin NPs for *in vitro* cellular imaging were studied using a confocal laser scanning microscope (CLSM). In these experiments, HeLa cervical cancer cells were incubated with TPE-TETRAD@biotin (6 μg mL^–1^) for 1 h at 37 °C. MitoTracker Green was chosen as a commercial reference to compare the photostability and bioimaging efficiency. For staining with MitoTracker Green, HeLa cells were incubated with 25 nM of MitoTracker Green for 10 min at 37 °C, as suggested by the manufacturer. The cellular uptake and colocalization of TPE-TETRAD@biotin and MitoTracker Green is shown in Fig. 4A–C. The tubular mitochondrial structure is clearly visible for all conditions and the image quality between TPE-TETRAD@biotin and MitoTracker Green is comparable. A high mitochondrial colocalization rate of 94.5% was achieved as calculated using the Leica DMI 6000 software (see ESI, Fig. S9†). The mitochondrial specificity is due to the receptor-mediated endocytosis pathway of the avidin–biotin interaction. Previous reports have shown that cells import biotinylated proteins through the avidin–biotin pathway to the mitochondria for amino acid catabolism.^45^ The cell is likely tricked into thinking that TPE-TETRAD@biotin NPs are biotinylated proteins and these are consequently trafficked to the mitochondria.

**Fig. 5:**
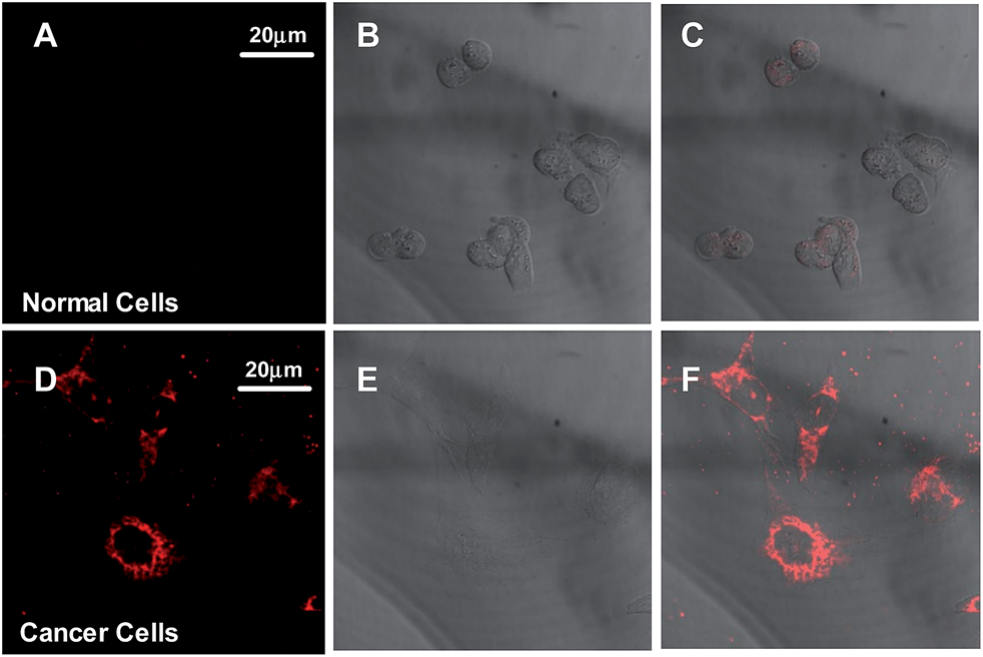
CLSM images of MDCK-II normal kidney cells (A–C) and HeLa cervical cancer cells (D–F) after incubation with 6 μg mL^–1^ TPE-TETRAD@biotin NPs for 1 h at 37 °C. (A and D) Fluorescent images, (B and E) bright field images and (C and F) merged images. *λ*_ex_ = 488 nm at 30% laser power, 600–750 nm emission filter, gain and offset held constant.

In order to evaluate the cellular targeting efficiency of the TPE-TETRAD@biotin NPs, a parallel control study was performed imaging MDCK-II non-cancerous kidney cells and HeLa cervical cancer cells. As shown in Fig. 5, CLSM images of MDCK-II non-cancerous kidney cells (A–C) show significantly less NP uptake and are consequently less bright when compared to HeLa cervical cancer cells (D–F) after incubation with 6 μg mL^–1^ TPE-TETRAD@biotin for 1 h at 37 °C, keeping all laser conditions constant. These results agree with previous literature showing that biotin can serve as a cost-effective cancer targeting moiety due to the overexpression of biotin binding sites on cancer cell surfaces when compared to normal cells.^44^

**Fig. 6:**
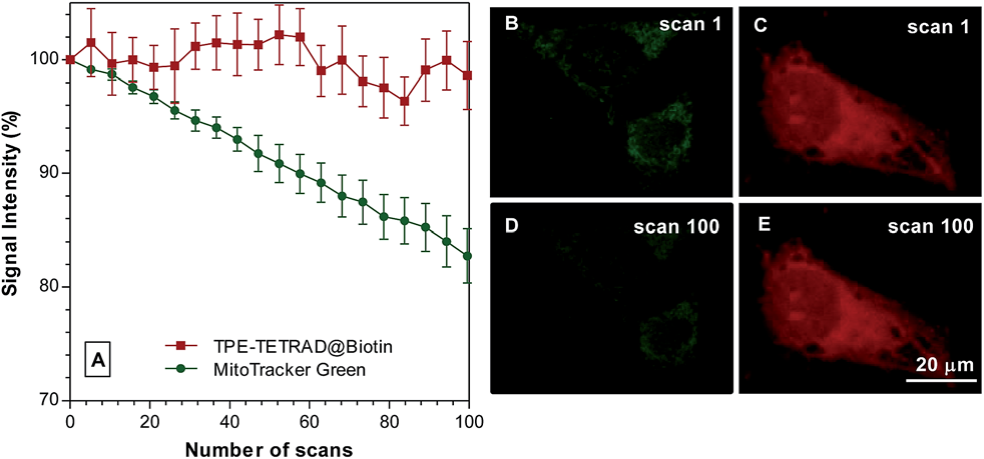
(A) Photostability comparison between TPE-TETRAD@biotin AIE NPs (red) and MitoTracker Green (green) in HeLa cervical cancer cells with increasing number of laser scans at 488 nm. Signal intensity is defined by *I*/*I*_0_, where *I*_0_ is the initial fluorescence intensity and *I* is the fluorescence intensity after each subsequent scan. (B–E) Fluorescent images at scan 1 and 100 for MitoTracker Green (green) and AIE NPs (red). Scale bar: 20 μm.

The photostability of the TPE-TETRAD@biotin NPs was tested against their closest commercial competitor, MitoTracker Green, *in vitro* using a CLSM. Images were recorded at 2 s intervals at an excitation wavelength of 488 nm at 30% laser power. MitoTracker Green is well known to be highly photostable when compared to similar commercial dyes such as Rhodamine 123 and MitoTracker Red. As shown in Fig. 6, TPE-TETRAD@biotin showed very little signal intensity decrease over a period of 100 scans, whereas MitoTracker Green had a 20% drop in signal intensity. The inset pictures in Fig. 6 show images at scan 1 and 100 for MitoTracker Green (green) and TPE-TETRAD@biotin (red). The high *in vitro* photostability of TPE-TETRAD@biotin is a direct result of its AIE characteristics.^48^ Previous reports have shown that high photostability is critical for *in vivo* two-photon imaging and multiphoton characterization.^57^ This result prompted us to investigate the high-order non-linear optical characteristics of TPE-TETRAD@biotin NPs.

### Multiphoton characterization and applications

**Fig. 7:**
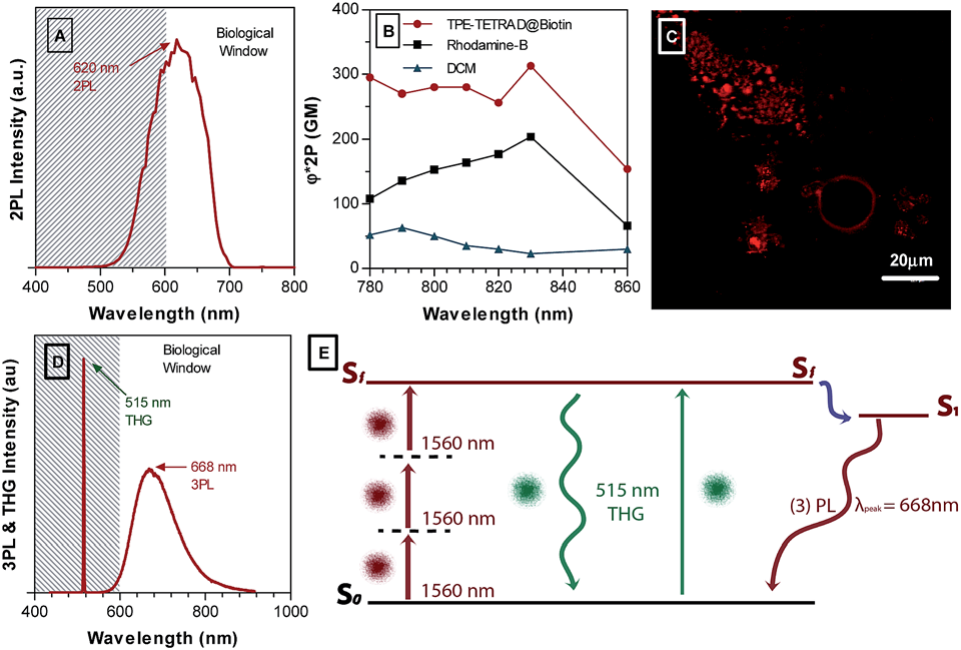
(A) Two-photon excitation fluorescence (TPEF) emission spectra for TPE-TETRAD@biotin NPs in water. (B) Two-photon absorption (2PA) cross-section of AIE NPs (red), Rhodamine B (black) and DCM (blue). (C) TPEF images of HeLa cervical cancer cells after incubation with 6 μg mL^–1^ AIE NPs for 1 h at 37 °C, using a multiphoton excitation wavelength of 980 nm. (D) Simultaneous three-photon luminescence and third harmonic generation (THG) emission spectra for AIE NPs excited by a femtosecond laser at 1560 nm. (E) Diagram showing the proposed excitation mechanism of THG and THG-induced photoluminescence.

Two-photon excitation fluorescence (TPEF) was used to characterize the two-photon absorption (2PA) cross-section of the TPE-TETRAD@biotin NPs. This is achieved by analyzing the two-photon emission intensity relative to a known standard such as Rhodamine B, where the exact 2PA is known for each wavelength measured according to well-known databases. Rhodamine B was chosen as a standard because its emission profile best matches that of TPE-TETRAD@biotin. Using a relative method with Rhodamine B, it was possible to calculate the 2PA cross-section for the AIE NPs, as shown in Fig. 7B.^61,62^ DCM is also included in Fig. 7B to emphasize the significant gains that were made in the 2PA cross-section *via* extending the π-conjugation length and D–A structure through synthetic modifications. A schematic illustration of the experimental setup is provided in Fig. S10, ESI.† The TPEF emission maximum is located at 620 nm, which is within the biological window illustrated in the spectra as the white region in Fig. 7A. High biocompatibility, photostability and a large 2PA cross-section ranging from 295 MG at 780 nm to its peak value of 313 at 830 nm indicates that TPE-TETRAD@biotin could be an effective probe for *in vivo* TPEF microscopy. To first test its efficacy *in vitro*, HeLa cervical cancer cells were incubated with 6 μg mL^–1^ TPE-TETRAD@biotin for 1 h at 37 °C and imaged using a TPEF microscope. As shown in Fig. 7C, the bright fluorescence signal of TPE-TETRAD can be used to clearly distinguish the cell profile, indicating that the AIE NPs can serve as promising two-photon fluorescent probes upon excitation at 980 nm.

Previous reports by our group have found that some AIE luminogens possess rich non-linear optical properties, including aggregation-induced third harmonic generation (THG) enhancement and aggregation-induced three-photon luminescence (3PL).^55^ A series of non-linear optical characterization experiments were performed to see if the TPE-TETRAD NPs possess similar characteristics.

The experimental setup is illustrated in Fig. S11, ESI.† Fig. 7D shows the simultaneous THG and 3PL for TPE-TETRAD NPs. The maximum 3PL emission occurs at 668 nm, which is well within the biological window for optical imaging. The 3PL emission is also significantly red-shifted relative to the 2PL and 1PL emission spectra. The THG peak occurs at 515 nm. Based on our previous report, we suggest an alternative mechanism for the generation of 3PL, as illustrated in Fig. 7E.^55^ We propose that THG photons are directly generated under femtosecond excitation and may be reabsorbed by the TPE-TETRAD molecules in NPs to produce fluorescence in a process known as THG-induced fluorescence. This may be a more efficient pathway to achieve 3PL signals, since direct 3PL is a fifth-order non-linear optical process, while 3PL induced by one-photon excitation of THG is a linear absorption based on a third-order NLO process. So far, this process is unique to AIE materials where an organic molecule simultaneously exhibits THG and 3PL in its aggregated state. In fact, previous reports have shown that the aggregation degree of AIEgens will lead to an increase in THG and THG-induced fluorescence in a process known as aggregation-induced THG enhancement.^63^ Future studies will be carried out to confirm if these characteristics hold true for TPE-TETRAD NPs. Furthermore, the bioimaging applications of TPE-TETRAD NPs based on 3PL and THG features will be explored in future studies.

## Conclusion

We applied a synthetic strategy to convert a TICT dye into a deep-red AIE-active fluorophore (TPE-TETRAD) with a favorable absorption and TICT + AIE features. TPE-TETRAD@biotin NPs were fabricated using a modified nanoprecipitation method and were found to selectively stain mitochondria in cancer cells. The AIE NPs demonstrated low cytotoxicity and low ROS generation abilities, making them promising candidates for long-term cell tracking. The AIE NPs possessed higher photostability than the commercial mitochondrial probe MitoTracker Green. Remarkably, the AIE NPs had high 2PA, favorable TPEF emission and produced quality TPEF cancer cell images. Furthermore, we verified that TPE-TETRAD NPs possess a rich array of non-linear optical properties including aggregation-induced THG enhancement and aggregation-induced 3PL. Future efforts will focus on using TPE-TETRAD@biotin NPs for multimodal non-linear optical imaging and 3PL *in vivo* imaging.

## Experimental methods

### Materials

The synthetic materials for the malonitrile derivative,^64^ TPA-DCM,^32^ TPE compounds^65^ and 2′,7′-dichlorofluorescin diacetate (DCFH-DA) were prepared according to previous synthetic reports from reagents obtained from Sigma-Aldrich. Cell culturing materials, including penicillin–streptomycin solution and 3-(4,5-dimethylthizaol-2-yl)-2,5-diphenylte-trazolium bromide (MTT), were purchased from ThermoFisher Scientific. Dulbecco’s Modified Eagle Medium (DMEM), modified essential medium (MEM) and MitoTracker Green (MTG) were purchased from Invitrogen. DSPE–PEG_2000_–biotin was purchased from Avanti Polar Lipids, Inc. Fetal bovine serum (FBS) and trypsin–EDTA solution were purchased from Life Technologies. Tetrahydrofuran (THF) and dichloromethane (DCM) were distilled from sodium benzophenone ketyl and calcium hydride under nitrogen, respectively. Milli-Q water was purified *via* the Milli-Q Plus System (Millipore Corporation). HeLa cervical cancer cells and MDCK-II noncancerous kidney cells were obtained from the American Culture Collection.

### Characterization

The UV-Vis absorption spectra were recorded using a Shimadzu UV-2600 spectrometer. The ^1^H and ^13^C-NMR spectra were recorded using a Bruker AV 400 spectrometer in CDCl_3_ using tetramethylsilane (*δ* = 0) as an internal reference. The photoluminescence (PL) spectra were recorded using a Perkin-Elmer LS 55 spectrofluorometer. The fluorescence lifetime was measured using a Hamamatsu Compact Fluorescence Lifetime Spectrometer C11367. The high-resolution mass spectra (HRMS) were recorded using a GCT premier CAB048 mass spectrometer operating in matrix-assisted laser desorption ionization time-of-flight (MALDI-TOF) mode. The particle size and the size distribution of the NPs were measured by DLS, using a 90Plus particle-size analyzer (Brookhaven Instruments Co.) at a fixed angle of 90° at room temperature. A Leica DMI 6000 fully motorized inverted microscope was used for CLSM and TPEF imaging. The morphology of the AIE NPs was investigated by TEM (JEM-2010F, JEOL, Japan).

### Synthesis

The synthetic strategies were based on well-established Knoevenagel condensation and Suzuki coupling reactions. The detailed synthetic route to TPE-TETRAD is illustrated in Scheme 2. The synthetic details to prepare TPA-DCM and 2Br-TPA (**2**) may be found in our previous report.^32^ The malononitrile derivative (**4**)^64^ and the TPE derivative (**1**)^65^ were prepared according to our previous reports. The 2TPE-TPA derivative (**3**) was prepared by mixing Pd(PPh_3_)_4_ (150 mg) as a catalyst, **1** (2.17 g, 5.8 mmol), **2** (833 mg, 0.1.9 mmol), and K_3_PO_4_ (1.5 g, 5 mmol) in 50 mL of THF and 8 mL of water under nitrogen. The mixture was heated to 70 °C for 36 h to yield a dark yellow mixture. After filtration and solvent evaporation under reduced pressure, the crude product **3** was purified by silica-gel column chromatography using hexane/dichloromethane as the eluent. The product yield was 50% yield (900 mg). Subsequently, piperidine (0.3 mL) was added into a solution of acetonitrile with **3** (198 mg, 0.2 mmol) and **4** (14.5 mg, 0.1 mmol). The mixture was heated to 100 °C for 72 h. After cooling to room temperature, the solution was extracted with dichloromethane (100 mL) twice, washed with water, and dried over Na_2_SO_4_. After filtration and solvent evaporation under reduced pressure, TPE-TETRAD was obtained as a red powder in 40% yield (200 mg) after silica-gel column chromatography purification using hexane/dichloromethane as the eluent. TPE-TETRAD has good solubility in common organic solvents such as tetrahydrofuran, toluene, dichloromethane and chloroform but is insoluble in water. TPE-TETRAD was characterized by ^1^H NMR, ^13^C NMR and high resolution mass spectrometry, which confirmed its correct chemical structure. ^1^H NMR (400 MHz, CDCl_3_), *δ* (ppm): 7.56–7.43 (m, 20H), 7.39–7.34 (m, 16H), 7.33–7.0 (m, 66H), 6.67 (d, *J* = 16 Hz, 2H; pyran –CH=), 6.63 (s, 2H; pyran H); ^13^C NMR (75 MHz, CDCl_3_), *δ* (ppm): 159.5, 150.0, 145.9, 143.8, 142.7, 141.2, 140.5, 139.1, 136.2, 131.6, 131.2, 128.7, 127.7, 126.2, 125.7, 122.1, 116.5, 115.2, 106.4, 53.4; HRMS (MALDI-TOF) for C_152_H_105_N_4_O: *m*/*z* 2003.8378 (M^+^, calculated 2004.5470).

### Fabrication of TPE-TETRAD@biotin NPs

The AIE NPs were prepared according to our previously published method. 1 mg mL^–1^ of TPE-TETRAD solution in THF (0.25 mL) and DSPE–PEG_2000_–biotin powder (2 mg) were mixed in a scintillation vial (20 mL).^57^ After adding 1 mL of THF, the mixture was stirred at room temperature for 30 min to form a homogeneous red solution and then was dried to a thin film with a gentle stream of N_2_. When the THF was completely removed, 1 mL of Milli-Q water was added into the vial and the solution was sonicated for 15 min (SCIENTZ-II D with 20% power for 10 s pulses with 1 s rests in between). The solution was then allowed to stir for an additional 1 h at room temperature. The solution was then filtered using a 0.45 μm filter. The TPE-TETRAD@biotin NP solution had a dried mass of roughly 0.25 mg with a concentration of 250 μg mL^–1^. The PEG/dye ratio leading to the greatest encapsulation efficiency was optimized based on our previous report.^32^ TPE-TETRAD@biotin NPs were stored in a scintillation vial at room temperature and were stable for several weeks.

### Cell culture

The HeLa cells were cultured in MEM while the MDCK-II cells were cultured in DMEM at 37 °C in a humidified incubator with 5% CO_2_. Both culture media contained 10% heat-inactivated FBS, 100 μg mL^–1^ penicillin and 100 μg mL^–1^ streptomycin. Before the experiments, the cells were pre-cultured until confluence was reached.

### Cell imaging

MDCK-II or HeLa cells were grown overnight on a 35 mm Petri dish with a cover slip. The live cells were stained with TPE-TETRAD@biotin (6 μg mL^–1^) for 1 h at 37 °C. For staining with MTG, the cells were incubated with 25 nM of MTG for 10 min at 37 °C, as suggested by Invitrogen. For co-staining experiments, the cells were first incubated with TPE-TETRAD@biotin (6 μg mL^–1^) for 1 h, then MTG (25 nM) for 10 min at 37 °C. Before imaging on the confocal microscope, the cells were washed with PBS three times. The treated cover-slips were then mounted in standard mounting media and imaged using a fluorescence microscope (Leica DMI 6000). The excitation wavelength was 488 nm for both TPE-TETRAD@biotin and MTG at 30% laser power. For TPE-TETRAD@biotin, a 600–750 nm emission filter was used. For MTG, a 500–580 nm emission filter was used. When comparing the staining efficiency between MDCK-II and HeLa cell lines, the laser power was held constant at 30% as well as all other laser parameters (digital gain and offset) to provide an accurate comparison. For two-photon excited fluorescence (TPEF) cell imaging, the same TPE-TETRAD@biotin sample preparation as used in the confocal imaging experiments was used. The pulsed multiphoton laser was activated at an optimized wavelength of 980 nm using the Leica DMI 6000 microscope. To study the photostability of TPE-TETRAD@biotin and MTG in cells, CLSM images were recorded at 2 s intervals with an excitation wavelength of 488 nm at 30% laser power. The fluorescence intensity of each image was analyzed using the Image Pro Plus software for each scan. The photostability of TPE-TETRAD@biotin and MTG was expressed by *I*/*I*_0_, where *I*_0_ is the initial fluorescence intensity and *I* is the fluorescence intensity after each subsequent scan. The data were obtained from replicated experiments (*n* = 20).

### Cytotoxicity and ROS generation of TPE-TETRAD@biotin AIE NPs

MTT assays were used to evaluate the cytotoxicity of TPE-TETRAD@biotin to the HeLa cells. The HeLa cells were seeded on 96-well plates (Costar) at a density of 1 × 10^4^ cells per well. The cells were exposed to a series of doses of TPE-TETRAD@biotin (0–100 μg mL^–1^) in culture medium at 37 °C. After 24 h incubation, 10 μL of freshly prepared MTT solution (5 mg mL^–1^ in PBS) was added into each well. After 4 h incubation, 100 μL of solubilizing solution containing 10% SDS and 0.01 M HCl was added to dissolve the MTT dye. After 8 h incubation, the absorbance of MTT at 595 nm was monitored using a Perkin-Elmer Victor plate reader. Cell viability was expressed by the ratio of absorbance of the cells incubated with TPE-TETRAD@biotin to that of cells incubated with culture medium only. The data were obtained from replicate experiments (*n* = 3). To evaluate the added cytotoxicity that may be generated from any reactive oxygen species produced by the dye NPs, a series of MTT assay control experiments were carried out. Following the procedure as above, replicate plates were prepared for two groups. One group was incubated under visible light excitation whereas the other group was kept under dark conditions. A DCFH-DA cellular reactive oxygen species detection assay kit (Sigma-Aldrich) was used to characterize the ROS generation from TPE-TETRAD@biotin and TPA-DCM.

### Multiphoton characterization

TPEF was used to determine the two-photon absorption (2PA) cross-section for TPE-TETRAD@biotin using a relative method with Rhodamine B as a standard.^61,62^ A mode-locked Ti:Sapphire oscillator (Coherent Mira 900, with a wavelength of 720–950 nm) was used to excite the sample with femtosecond pulses with a repetition rate of 76 MHz. The detector consisted of a SpectraPro275 spectrometer coupled to a photomultiplier tube (PMT) with a lock-in amplifier system to enhance the signal-to-noise ratio. The optical components consisted of a Semrock 670 nm edge bright line multiphoton short-pass dichroic beam splitter, 10× NA = 0.25 objective lens and 2 mm thick quartz sample cell (see ESI, Fig. S10†). The TPEF and 2PA cross-sections were estimated using a previously published method.^66^ A calibration curve was constructed for the TPE-TETRAD NPs in order to check the concentration and make sure a homogeneously dispersed suspension was used. The 2PA properties were studied in the wavelength range of 780–860 nm using a Ti:Sapphire oscillator. The experimental set-up of the non-linear optical microscopic system is shown in Fig. S11, ESI.† A 1560 nm fs laser beam (FLCPA-01C, Calmar Laser, 1 MHz, 400 fs) was coupled into an upright confocal microscope (BX61 + FV1000, Olympus). The beam was reflected by DC1 (dichroic mirror: 1000–1600 nm reflection, 400–950 nm transmission, customized by Chroma Technology Corp), and then raster-scanned by two galvanometer-driven mirrors. Scan mirrors were imaged onto the back aperture of the microscope objective by a scan lens and a tube lens. A water-immersed microscope objective (XLPLN25XWMP2, Olympus, 25 × 1.05 NA) was used to focus the excitation beam into the sample and to epi-collect the three-photon luminescence (3PL) and third harmonic generation (THG) signals. Epi-collected 3PL and THG signals (wavelength < 900 nm) passed through the tube lens, scan lens, two galvanometer-driven mirrors, DC1 and a pinhole (size: 800 μm), and recorded with an optical fibre spectrometer (PG 2000, Ideaoptics Instruments). During the measurement, glass capillary tubes filled with TPE-TETRAD NPs in aqueous solution were put under the objective, and deuteroxide was smeared between the water-immersed objective and the top glass surface of the capillary tubes.
