# Supplementary material for: Functionalized AIE nanoparticles with efficient deep-red emission, mitochondrial specificity, cancer cell selectivity and multiphoton susceptibility
Source: Chem Sci. 2017 May 9;8(6):4634–43. doi: 10.1039/c7sc00908a (PMC5618339; doi:10.1039/c7sc00908a)
Supplement: Supplementary file 1 [file SC-008-C7SC00908A-s001.pdf]

Electronic Supplementary Material (ESI) for Chemical Science.
This journal is © The Royal Society of Chemistry 2017

Supporting Information

Functionalized AIE nanoparticles with efficient deep-red emission, mitochondria specificity, cancer cell selectivity and multiphoton susceptibility

Alexander Nicol,[ab] Wei Qin,[ab] Ryan T. K. Kwok,[ab] Jeffery Mark Burkhartsmeyer,[c] Zhenfeng Zhu,[d] Huifang Su,[ab] Wenwen Luo,[e] Jacky W. Y. Lam,[ab] Jun Qian,[d] Kam Sing Wong,[c] and Ben Zhong Tang*[abe]

[a] *Division of Biomedical Engineering, Department of Chemistry, Hong Kong Branch of Chinese National Engineering Research Center for Tissue Restoration and Reconstruction, Institute for Advanced Study, Institute of Molecular Functional Materials and State Key Laboratory of Molecular Neuroscience, The Hong Kong University of Science and Technology (HKUST), Clear Water Bay, Kowloon, Hong Kong, China. Email:* tangbenz@ust.hk

[b] *HKUST-Shenzhen Research Institute No. 9 Yuexing 1st RD, South Area, Hi-tech Park Nanshan, Shenzhen 518057, China.*

[c] *Department of Physics, HKUST, Clear Water Bay, Kowloon, Hong Kong, China*

[d] *State Key Laboratory of Modern Optical Instrumentation, Centre for Optical and Electromagnetic Research, Zhejiang Provincial Key Laboratory for Sensing Technologies, Zhejiang University 310058 Hangzhou, China Guangdong Innovative Research Team, SCUT-HKUST Joint Research Laboratory, State Key Laboratory of Luminescent Materials and Devices, South China University of Technology, Guangzhou 510640, China*

[e] *Guangdong Innovative Research Team, SCUT-HKUST Joint Research Laboratory, State Key Laboratory of Luminescent Materials and Devices, South China University of Technology, Guangzhou 510640, China*

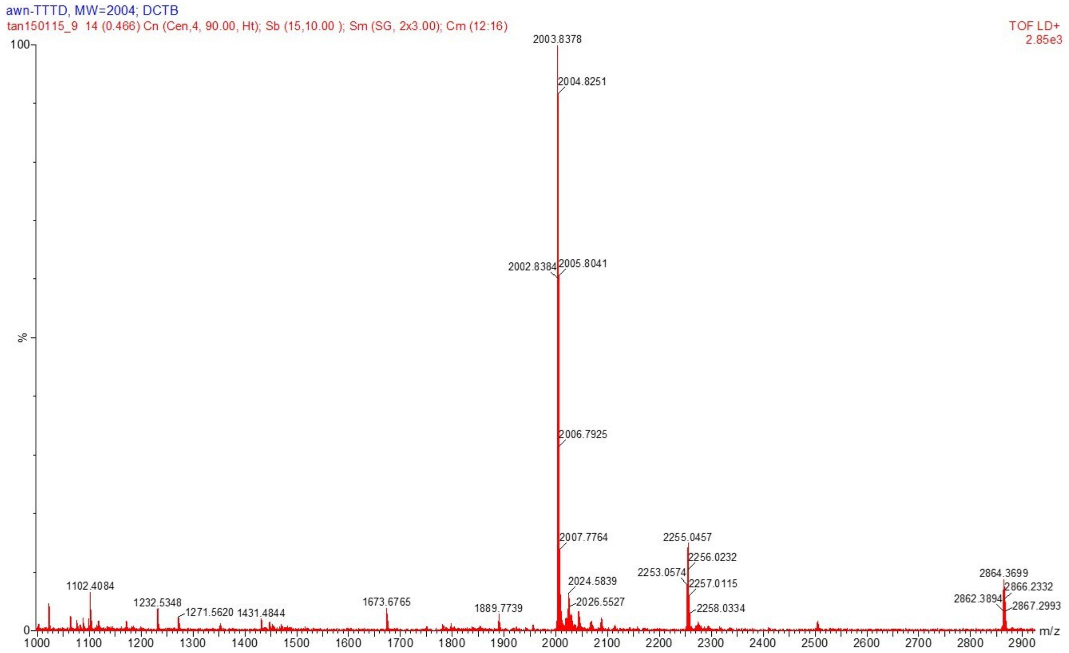

Figure S1. HRMS spectrum of TPE-TETRAD.

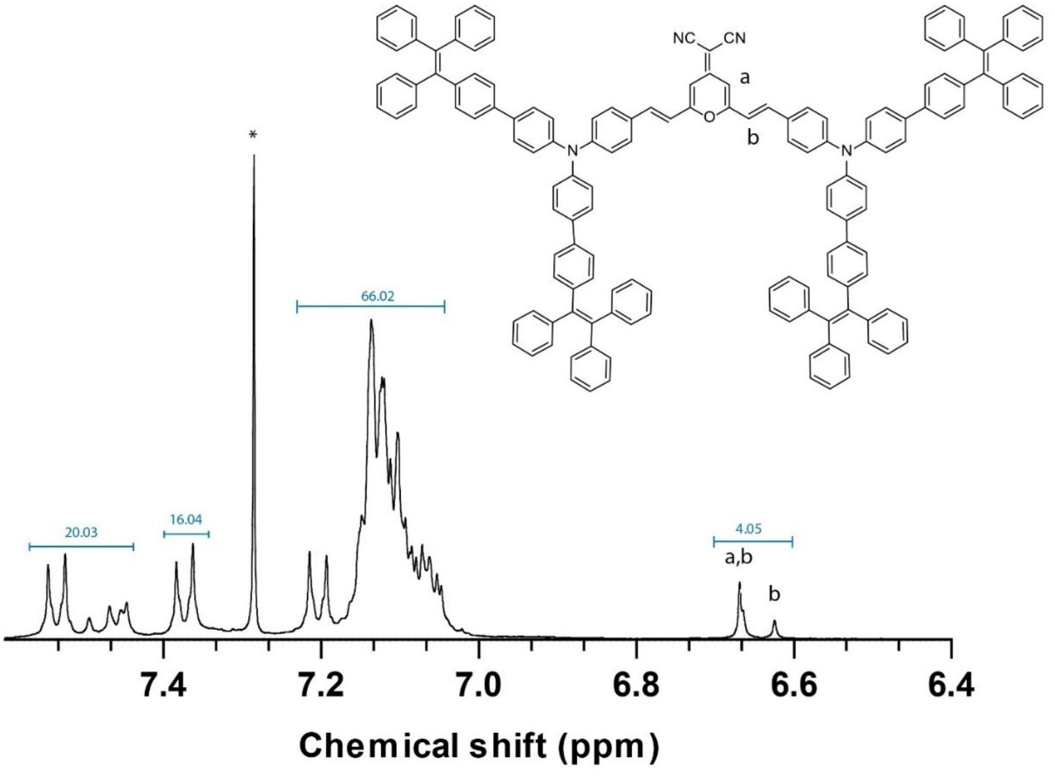

Figure S2. ^{1}H NMR spectrum of TPE-TETRAD. ^{1}H NMR (400 MHz, $CDCl_3$), δ (ppm): 7.56–7.43 (m, 20H), 7.39–7.34 (m, 16H), 7.33–7.0 (m, 66H), 6.67 (d, J = 16 Hz, 2H; pyran –CH=), 6.63 (s, 2H; pyran H).

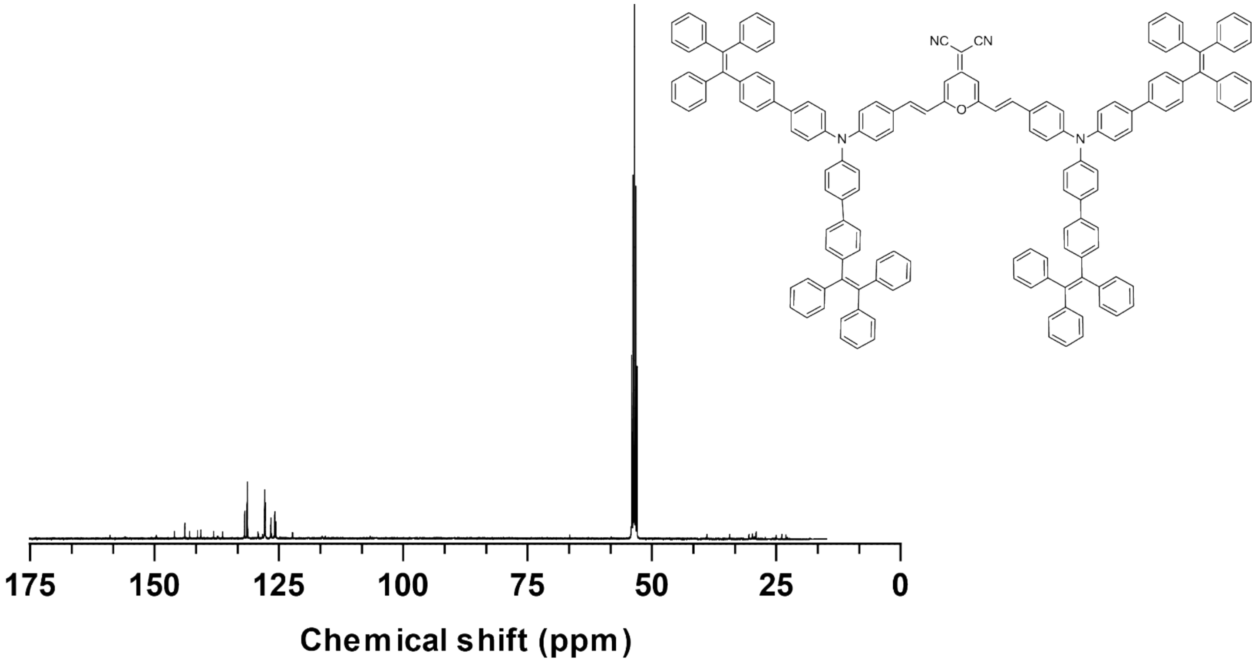

Figure S3. ^{13}C NMR spectrum of TPE-TETRAD. ^{13}C NMR (75 MHz, $CDCl_3$), δ (ppm): 159.5, 150.0, 145.9, 143.8, 142.7, 141.2, 140.5, 139.1, 136.2, 131.6, 131.2, 128.7, 127.7, 126.2, 125.7, 122.1, 116.5, 115.2, 106.4, 53.4.

Table S1. Optical Properties of TPA-DCM and TPE-TETRAD

	λ_{ab} (nm)[a]	E_g (eV)[b]	λ_{em} (nm)[c]		Stokes shift[d]
			solution	aggregate	
TPA-DCM	465	2.72	620	670	155
TPE-TETRAD	500	2.56	668	675	168

a)Absorption maxima (λ_{ab}) in THF(10 μM); b)HOMO-LUMO band gap (E_g) derived from theoretical DFT calculations; c)emission maxima (λ_{em}) for solution (in THF,10 μM) and aggregate (in THF/water 1:9 v/v, 10 μM); d)Stokes shift derived from subtracting λ_{em} from λ_{ab}.

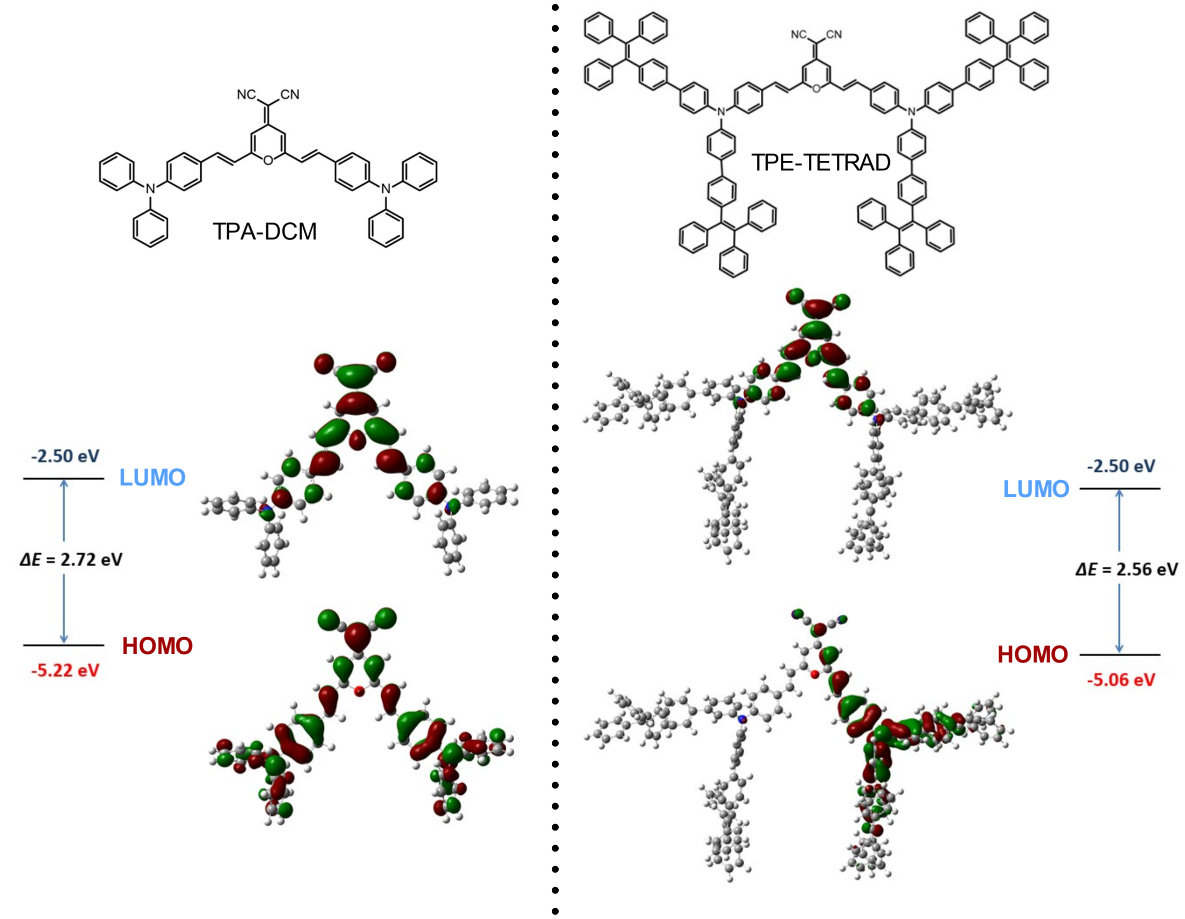

Figure S4. HOMO and LUMO energy levels of TPA-DCM and TPE-TETRAD, respectively. Molecular orbital amplitude plots of HOMO and LUMO energy levels of TPA-DCM and TPE-TETRAD were calculated using density functional theory (DFT) in Gaussian 09 program using B3LYP/6-31G(d) basis set.

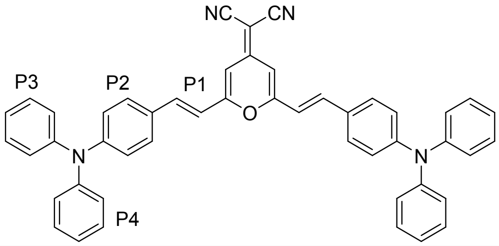

Angles between planes	
P_1-P_2	~0
P_2-P_3	~67
P_2-P_4	~67
P_3-P_4	~76

Figure S5. Summary of dihedral angles (°) for TPA-DCM. The dihedral angles between any two phenyl rings of TPA in TPA-DCM are from ~67° to ~76°.

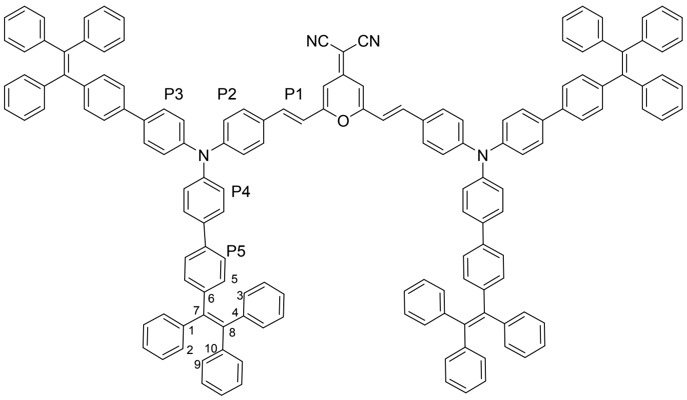

Dihedral angles (°)	
C5-C6-C7-C8	-48.4
C3-C4-C7-C8	-49.9
C2-C1-C7-C8	-50.0
C9-C10-C8-C7	-49.7

Angles between planes	
P_1-P_2	~0
P_2-P_3	~68
P_2-P_4	~67
P_3-P_4	~75
P_4-P_5	~35

Figure S6. Summary of dihedral angles (°) for TPE-TETRAD. In the TPE component of TPE-TETRAD, the dihedral angles between any phenyl rings are ~50°. The dihedral angles between any two phenyl rings of TPA in TPE-TERAD are from ~67° to ~75°.

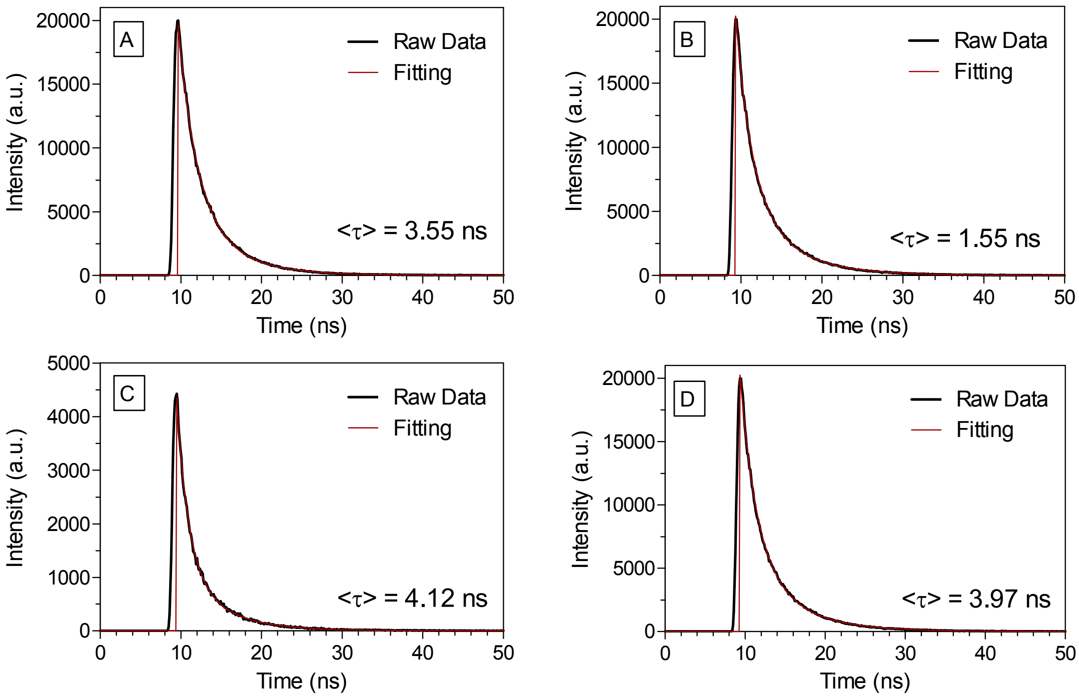

Figure S7. Fluorescence lifetime of (A) TPE-TETRAD thin film solid, (B) TPE-TETRAD nanoaggregates (10 μM) in water, (C) TPE-TETRAD@Biotin NPs (6 μg/mL) in water and (D) TPE-TETRAD (10 μM) in THF.

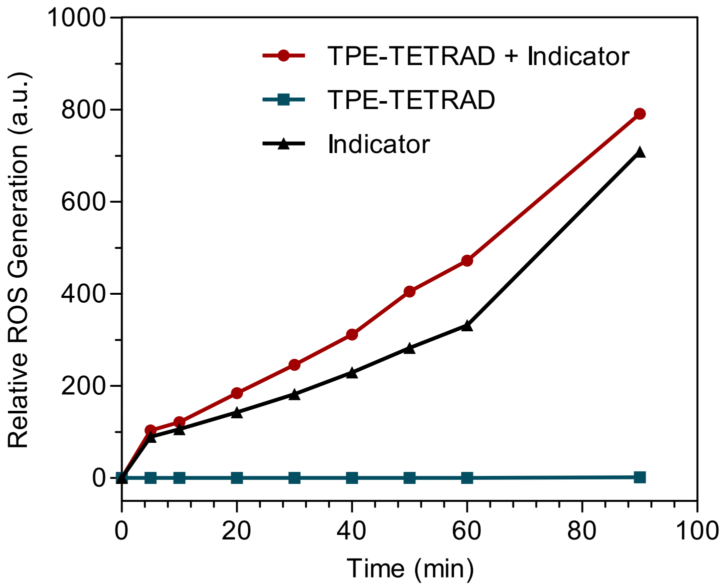

Figure S8. Relative ROS generation profile for TPE-TETRAD using DCFH-DA cellular reactive oxygen species detection assay kit. Change in fluorescent intensity at 534 nm for TPE-TETRAD, DCFH-DA indicator and their mixture in PBS upon white light irradiation for various time intervals. Excitation wavelength: 485 nm. [TPE-TETRAD] = 10 μM; [DCFH-DA] = 1 μM.

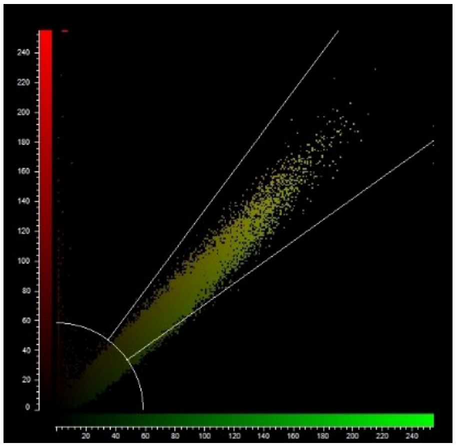

Colocalization Statistics

Pearson's Correlation	0.919
Overlap Coefficient	0.9288
Colocalization Rate	94.53%
Colocalization Area	306.53 ÂµmÂ²
Area Image	5302.86 ÂµmÂ²
Area Foreground	324.27 ÂµmÂ²
Area Background	4978.70 ÂµmÂ²

Figure S9. CLSM colocalization statistics for HeLa cervical cancer cells stained with 25 nM MitoTracker Green for 10 min and 6 µg/mL TPE-TETRAD@Biotin NPs for 1 h at 37 °C.

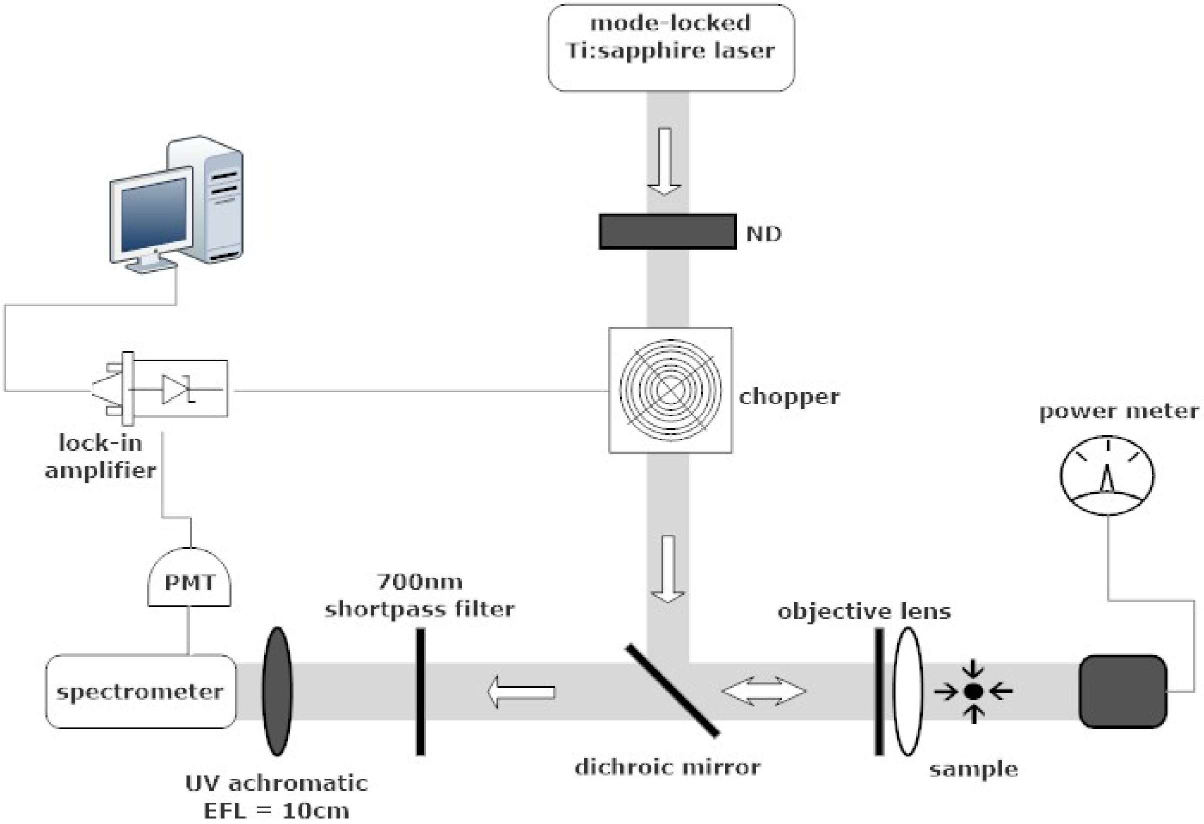

Figure S10. Schematic illustration of the experimental setup for two-photon excited fluorescence.

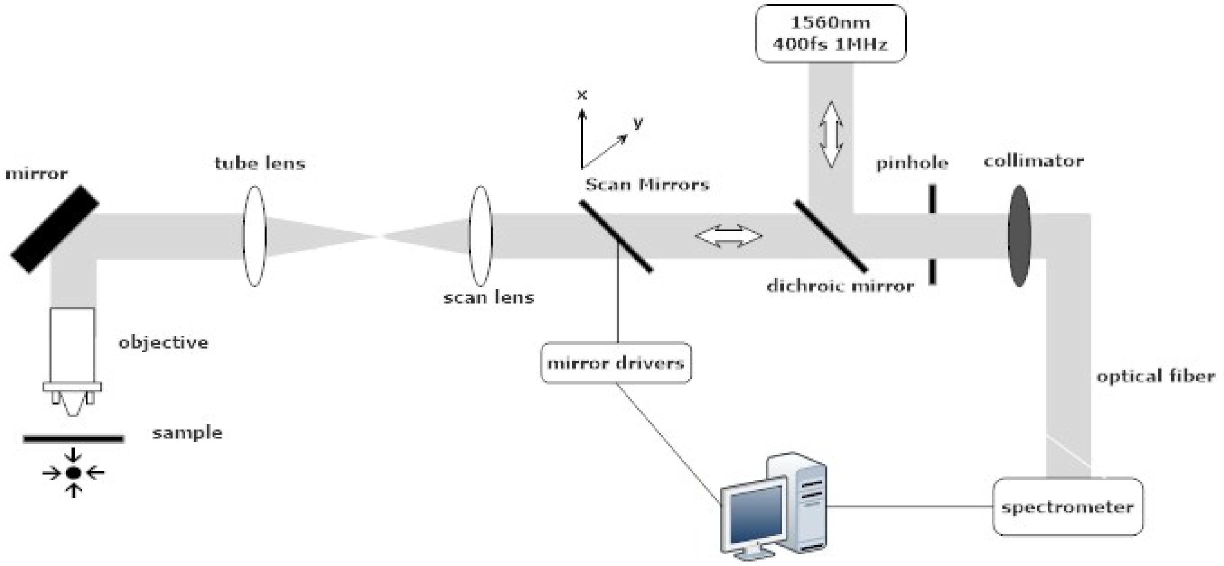

Figure S11. Schematic illustration of the NLO microscope system for spectrum measurement.

Calculation of TPE-TETRAD@Biotin AIE NPs Concentration

The density of TPE-TETRAD@Biotin can be estimated as 1 g/cm^3 due to its high water stability. The average size of the AIE NPs as verified by TEM and DLS was 100 nm. 0.25 mg of powder was obtained after freeze-drying 1 mL of the filtered stock solution. The TPE-TETRAD@Biotin concentration in a 1 mL stock suspension can be calculated from the following equation:

$$\frac{Total\ volume\ of\ TPE-TETRAD@Biotin\ NP}{Average\ volume\ of\ each\ NP} = \frac{\frac{0.25\ x\ 10^{-3}}{1g/mL}}{\frac{4}{3}\pi\ x\ (50\ x\ 10^{-7})^3 mL} = 4.78\ x\ 10^{11}$$

, Total number of NP in 1mL.

$$[TPE-TETRAD@Biotin] = \frac{\frac{4.78\ x\ 10^{11}}{6.02\ x\ 10^{23}\ mol^{-1}}}{1\ x\ 10^{-3}L} = 0.8\ nM$$

, final concentration in stock solution

$$Working\ Concentration = \frac{0.8nM\ x\ 50\mu L}{2000\mu L} = 20\ pM$$

, final working concentration for cell imaging

Or alternatively we can calculate the mass concentration:

$$Working\ Concentration = \frac{250\mu g/mL\ x\ 50\mu L}{2000\mu L} = 6.25\ \mu g/mL$$

, final working concentration for cell imaging.
